# From Lincoln to Cleveland, and Other Studies in History and General Literature

**Published:** 1886-03

**Authors:** 


FROM LINCOLN TO CLEVELAND, AND OTHER STUDIES IN HISTORY AND GENERAL LITERATURE. By Rev. W. J. Scott. Jas. P. Harrison & Co., Atlanta, Ga., 1886.

This new volume from a Southern press and by a popular Southern author will be sought after with great eagerness.

The description of the two types of civilization on this continent is masterly in matter and style. This feature is not only strikingly original, but exceedingly forcible. The other essays are delightfully written and will be richly enjoyed.

